# Long noncoding RNA POU6F2-AS2 contributes to the aggressiveness of nonsmall-cell lung cancer via microRNA-125b-5p-mediated E2F3 upregulation

**DOI:** 10.18632/aging.204639

**Published:** 2023-04-06

**Authors:** Haitao Yang, Xiao Feng, Xiangdong Tong

**Affiliations:** 1 Department of Thoracic Surgery, The People’s Hospital of Liaoning Province, Liaoning 110016, P.R. China

**Keywords:** POU6F2 antisense RNA 2, E2F transcription factor 3, microRNA, NSCLC progression

## Abstract

The role of the majority of long noncoding RNAs (lncRNAs) in the progression of nonsmall-cell lung cancer (NSCLC) remains elusive, despite their potential value, thus warranting in-depth studies. For example, detailed functions of the lncRNA POU6F2 antisense RNA 2 (POU6F2-AS2) in NSCLC are unknown. Herein, we investigated the expression status of POU6F2-AS2 in NSCLC. Furthermore, we systematically delineated the biological roles of POU6F2-AS2 in NSCLC alongside its downstream molecular events. We measured the expression levels of POU6F2-AS2 using quantitative real-time polymerase chain reaction and performed a series of functional experiments to address its regulatory effects in NSCLC cells. Using bioinformatic platforms, RNA immunoprecipitation, luciferase reporter assays, and rescue experiments, we investigated the potential mechanisms of POU6F2-AS2 in NSCLC. Subsequently, we confirmed the remarkable overexpression of POU6F2-AS2 in NSCLC using The Cancer Genome Atlas database and our own cohort. Functionally, inhibiting POU6F2-AS2 decreased NSCLC cell proliferation, colony formation, and motility, whereas POU6F2-AS2 overexpression exhibited contrasting effects. Mechanistically, POU6F2-AS2 acts as an endogenous decoy for microRNA-125b-5p (miR-125b-5p) in NSCLC that causes the overexpression of the E2F transcription factor 3 (E2F3). Moreover, suppressing miR-125b-5p or increasing E2F3 expression levels sufficiently recovered the anticarcinostatic activities in NSCLC induced by POU6F2-AS2 silencing. Thus, POU6F2-AS2 aggravates the oncogenicity of NSCLC by targeting the miR-125b-5p/E2F3 axis. Our findings suggest that POU6F2-AS2 is a novel therapeutic target for NSCLC.

## INTRODUCTION

Lung cancer is one of the most commonly diagnosed types of cancer and poses a major threat to health worldwide [1]. Annually, ~2.1 million new lung cancer cases are reported globally with >1.8 million mortalities [2]. Nonsmall-cell lung cancer (NSCLC) constitutes the predominant histological subtype, accounting for ~85% of all lung cancers, and is characterized by high aggressiveness and poor prognosis [3]. Despite marked improvement in treatment strategies, including surgical excision, radiochemotherapy, and immunotherapy, the clinical outcome of NSCLC remains poor [4]. The 5-year overall survival rate of patients with NSCLC is ~20%, partially because most cases are diagnosed at advanced stages [5]. Moreover, the complicated pathogenesis of NSCLC and absence of a promising therapeutic target are considered major obstacles preventing better clinical outcomes [6]. Therefore, there is an urgent need to discover the detailed mechanisms underlying NSCLC initiation and progression to identify and evaluate new therapeutic targets for NSCLC.

Long noncoding RNAs (lncRNAs) comprise a group of RNA molecules that are >200 nucleotides in length [7]. Although they do not act as templates for protein biosynthesis, their functions in biological and pathological processes have been extensively investigated [8]. Recently, numerous studies have implicated the dysregulation of lncRNAs in almost all types of human diseases, including cancer [9–11]. Reportedly, NSCLC involves the aberrant expression of several lncRNAs, and their dysregulation contributes to the oncogenesis of NSCLC [12–14]. For instance, an lncRNA named SNHG7 aggravates the malignancy and promotes the cisplatin resistance of NSCLC [15]. Furthermore, the ablation of the lncRNA DLGAP1-AS1 decreases the cell viability and motility of NSCLC cells and promotes their apoptosis [16]. LncRNAs perform pro-oncogenic or antioncogenic regulatory activities and play unique roles in mediating cell biological functions [17].

MicroRNAs (miRNAs) are a family of regulatory single-stranded RNA molecules with a transcript length of 17–22 nucleotides [18]. Although they do not encode proteins, numerous studies have highlighted their major contribution to NSCLC genesis and progression [19]. Recently, a competing endogenous RNA (ceRNA) network has been proposed and reported to exhibit considerable regulatory activity in human cancers [20]. LncRNAs sequester miRNAs, forming a ceRNA pattern that modulates the expression levels of miRNA target genes [21]. Thus, the ceRNA pathway is an attractive and potential therapeutic target for NSCLC.

Data from The Cancer Genome Atlas (TCGA) database indicated the overexpression of POU6F2 antisense RNA 2 (POU6F2-AS2) in lung adenocarcinoma (LUAD) and lung squamous cell carcinoma (LUSC). However, the detailed functions of POU6F2-AS2 in NSCLC remain unexplored. Herein, we investigated the expression status of POU6F2-AS2 in NSCLC. Furthermore, we systematically explored the biological roles of POU6F2-AS2 in NSCLC alongside its downstream molecular events. Our data confirmed that the POU6F2-AS2/miR-125b-5p/E2F transcription factor 3 (E2F3) axis plays a major role in NSCLC progression, suggesting that it can be a therapeutic target for managing NSCLC.

## RESULTS

### POU6F2-AS2 promotes the malignant phenotype of NSCLC cells

**Figure 1:**
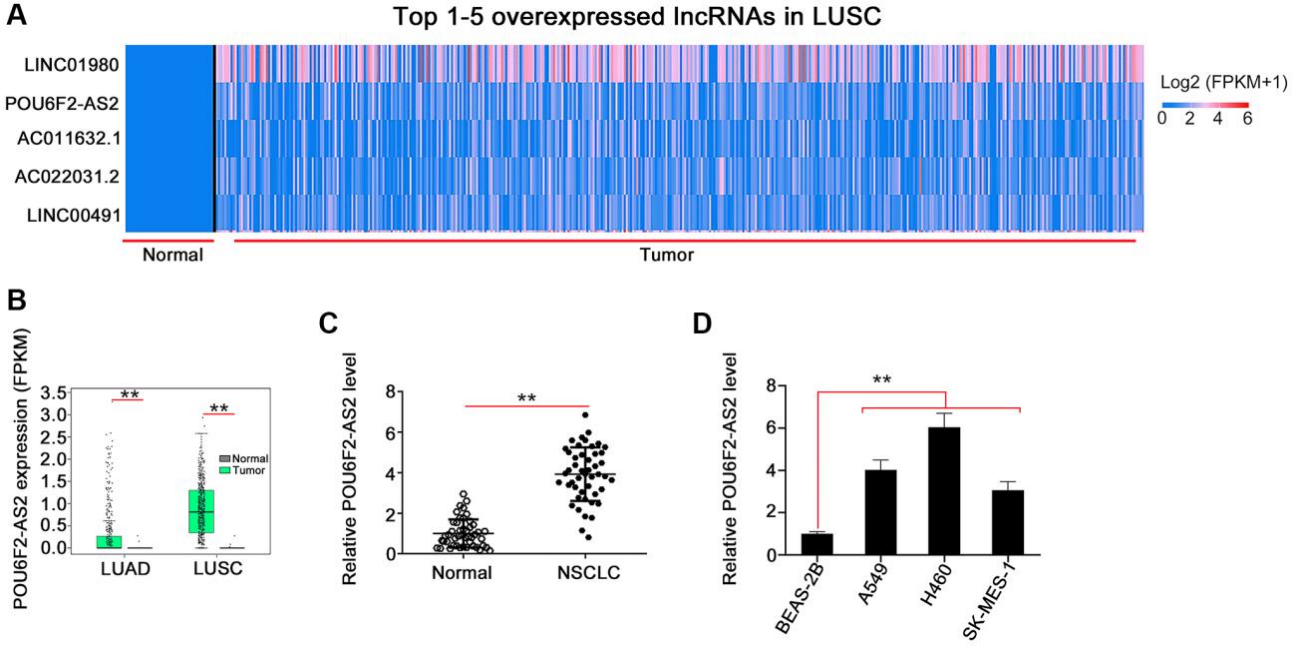
**POU6F2-AS2 is overexpressed in NSCLC.** (**A**) POU6F2-AS2 ranks the 2nd overexpressed lncRNA in LUSC. (**B**) POU6F2-AS2 level in LUAD and LUSC from TCGA database. ^**^P < 0.001 vs. normal group. (**C**) POU6F2-AS2 level in NSCLC tissues compared with normal tissues from our own cohort. ^**^P < 0.001 vs. normal group. (**D**) POU6F2-AS2 level in NSCLC cell lines. ^**^P < 0.001 vs. BEAS2-2B.

To investigate the expression pattern of lncRNAs in human cancers, we initially analyzed the TCGA database, which showed that POU6F2-AS2 was the second most overexpressed lncRNA in LUSC (Figure 1A). Furthermore, we found remarkably elevated expression levels of POU6F2-AS2 in LUAD and LUSC (Figure 1B). To validate this observation, we performed real-time quantitative polymerase chain reaction (RT-qPCR) to determine the expression levels of POU6F2-AS2 in NSCLC tissues and the adjacent nontumorous tissues obtained from our cohort, which confirmed the upregulation of POU6F2-AS2 in NSCLC tissues (Figure 1C).

**Figure 2:**
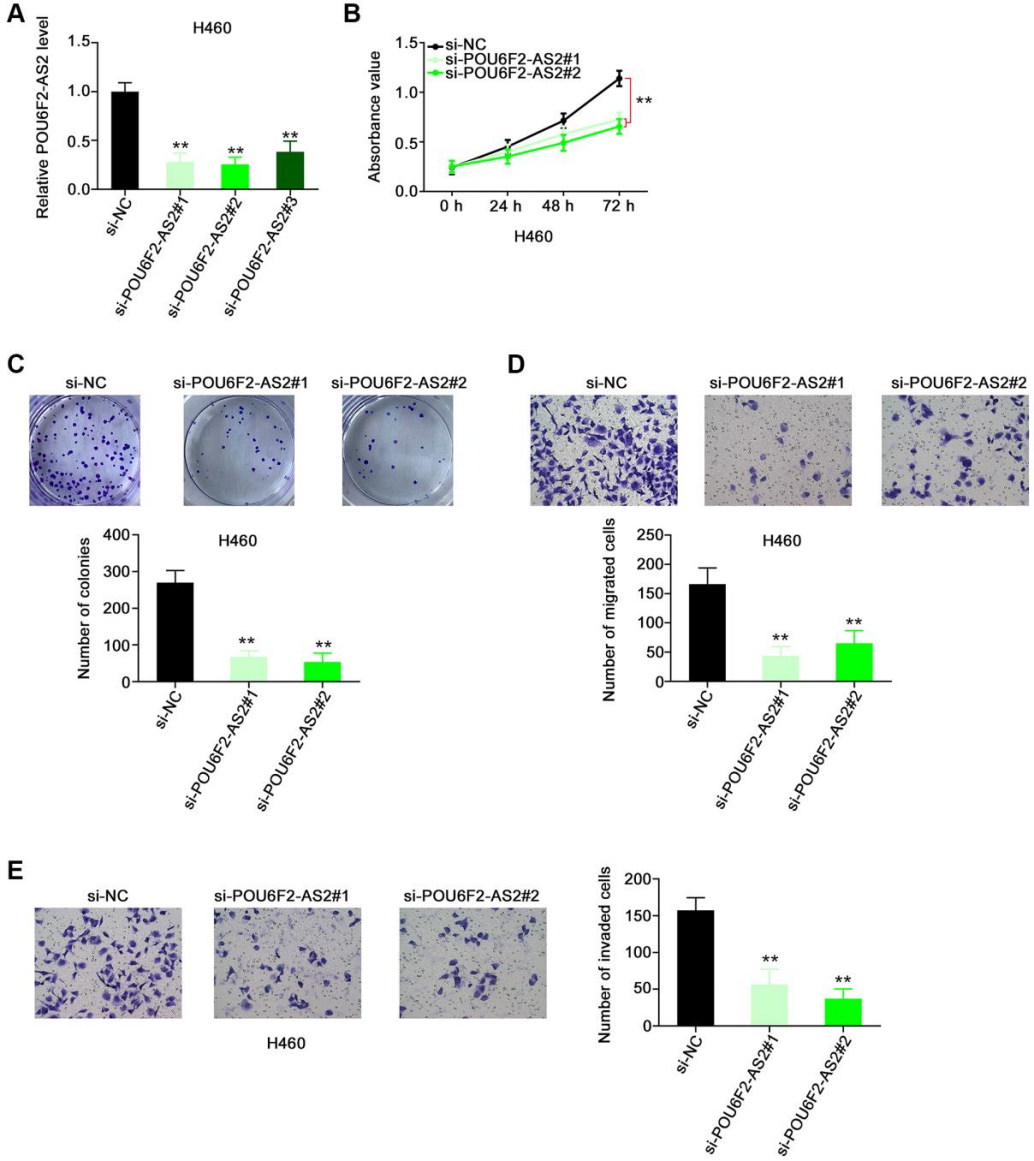
**Disturbing POU6F2-AS2 expression hampers the malignant phenotype of NSCLC cells.** (**A**) The knockdown efficiency of si-POU6F2-AS2 in H460 cells was uncovered by qRT-PCR. ^***^P < 0.001 vs. si-NC group. (**B** and **C**) The proliferation and colony-forming of POU6F2-AS2-silenced H460 cells. ^**^P < 0.001 vs. si-NC group. (**D** and **E**) The motility of H460 cells after POU6F2-AS2 downregulation. ^**^P < 0.001 vs. si-NC group.

Considering the upregulation of POU6F2-AS2 in NSCLC, we examined its regulatory activity in NSCLC progression. First, we explored the expression pattern of POU6F2-AS2 in NSCLC cell lines. All NSCLC cell lines exhibited higher expression levels of POU6F2-AS2 than nontumorigenic BEAS-2B cells (Figure 1D). Particularly, among all the NSCLC cell lines, the elevated expression levels of POU6F2-AS2 were considerably more pronounced in the H460 cell line, which was selected for the subsequent loss-of-function experiments. Three small interfering RNAs (siRNAs) were designed to selectively knockdown POU6F2-AS2 in NSCLC cells, and their silencing efficacy was assessed via RT-qPCR. Subsequently, two siRNAs, si-POU6F2-AS2#1 and si-POU6F2-AS2#2, were selected for the loss-of-function experiments owing to their better silencing efficacy (Figure 2A). The Cell Counting Kit-8 (CCK-8) and colony formation assays revealed that POU6F2-AS2 downregulation impeded NSCLC cell proliferation (Figure 2B and 2C). Additionally, transwell migration and invasion assays revealed the limited migratory and invasive abilities of cells treated with si-POU6F2-AS2#1 or si-POU6F2-AS2#2 (Figure 2D and 2E).

**Figure 3:**
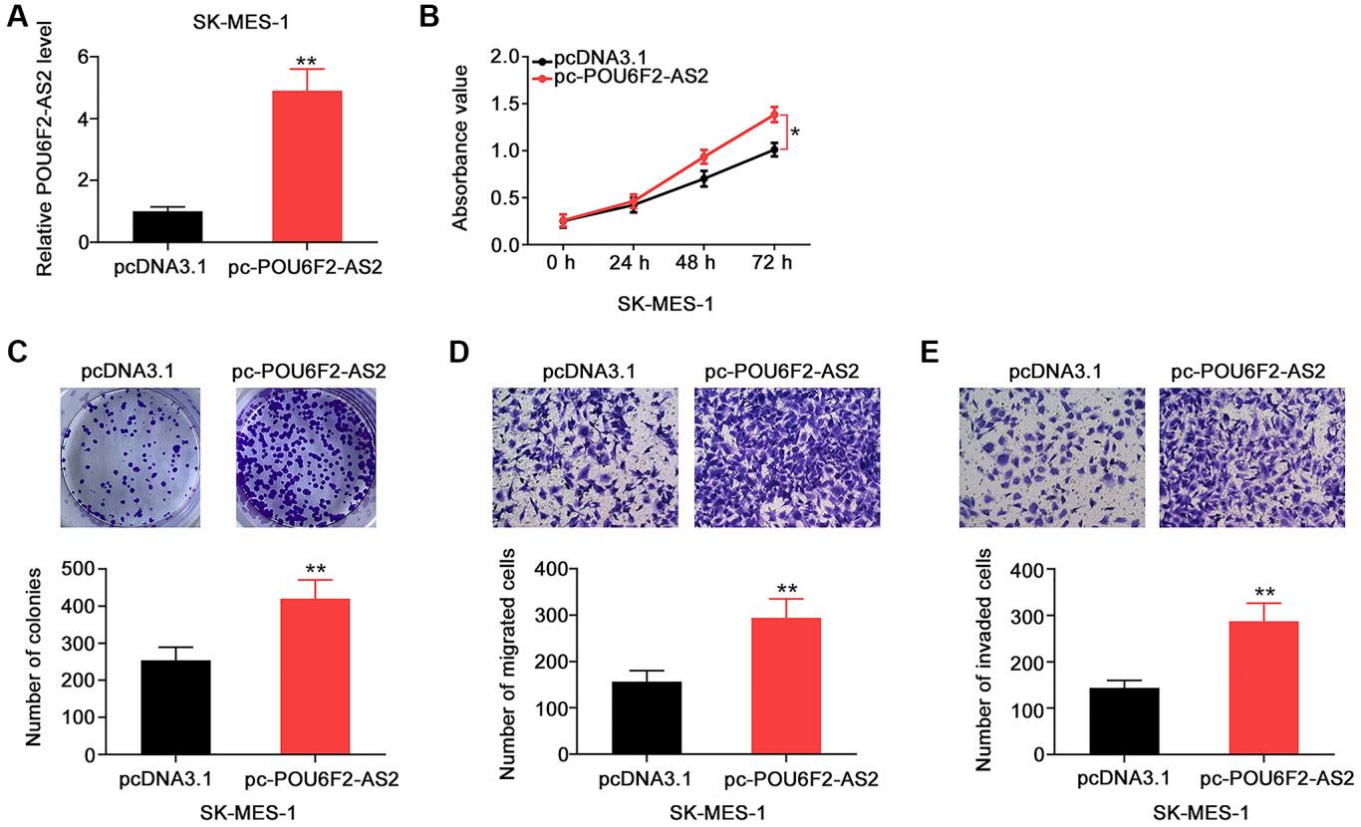
**POU6F2-AS2 upregulation promotes the aggressiveness of NSCLC cells.** (**A**) The transfection efficiency of pc-POU6F2-AS2 in SK-MES-1 cells. ^**^P < 0.001 vs. pcDNA3.1 group. (**B** and **C**) The proliferation and colony formation of POU6F2-AS2-overexpressed SK-MES-1 cells. ^*^P < 0.01 vs. pcDNA3.1 group. ^**^P < 0.001 vs. pcDNA3.1 group. (**D** and **E**) The motility of SK-MES-1 cells after POU6F2-AS2 upregulation. ^**^P < 0.001 vs. pcDNA3.1 group.

Furthermore, the overexpression of POU6F2-AS2 with pc-POU6F2-AS2 in SK-MES-1 cells (Figure 3A) enhanced the proliferative (Figure 3B) and colony-forming (Figure 3C) abilities of SK-MES-1 cells. Additionally, POU6F2-AS2 overexpression promoted the motility of SK-MES-1 cells (Figure 3D and 3E). Thus, POU6F2-AS2 functions as a tumor-promoting factor in NSCLC.

### POU6F2-AS2 acts as a sponge for miR-125b-5p in NSCLC

**Figure 4:**
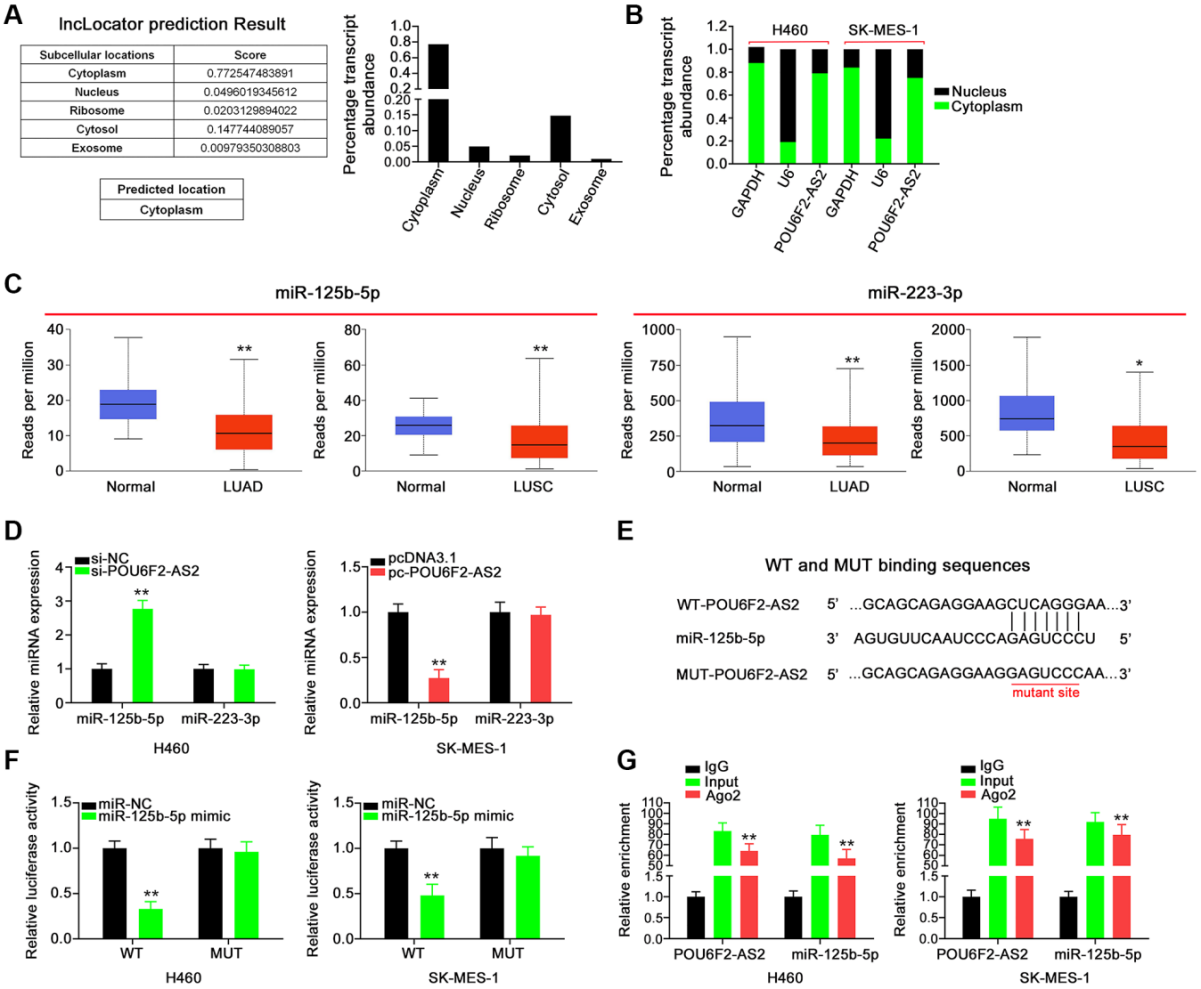
**POU6F2-AS2 executes as a miR-125b-5p sponge in NSCLC.** (**A**) The location of POU6F2-AS2 predicted by lncLocator. (**B**) The detection of the POU6F2-AS2’s subcellular location by nuclear–cytoplasmic fractionation assay. (**C**) Expression of miR-125b-5p and miR-223-3p in LUAD and LUSC samples from TCGA database. ^*^P < 0.01 and ^**^P < 0.001 vs. normal group. (**D**) Expression of the aforementioned candidates in NSCLC cells after disturbing POU6F2-AS2 level. ^**^P < 0.001 vs. si-NC group. (**E**) The binding sequences between POU6F2-AS2 and miR-125b-5p. (**F**) Luciferase activity induced by WT-POU6F2-AS2 or MUT-POU6F2-AS2 was examined in NSCLC after miR-NC or miR-125b-5p mimic transfection. ^**^P < 0.001 vs. miR-NC group. (**G**) RIP experiment corroborated the interaction between POU6F2-AS2 and miR-125b-5p. ^**^P < 0.001 vs. IgG group.

To delineate the mechanisms through which POU6F2-AS2 potentially affects tumor progression, we examined the subcellular localization of POU6F2-AS2 in NSCLC cells. Using the bioinformatics tool lncLocator, we predicted that POU6F2-AS2 was distributed in the cytoplasm (Figure 4A), which was further confirmed via nuclear–cytoplasmic fractionation assay in NSCLC cells (Figure 4B). This implies that POU6F2-AS2 exerts its regulatory effects by acting as miRNA sponge. Using miRcode, we predicted 10 miRNAs that exhibited high binding potential to POU6F2-AS2 (Table 1), among which two miRNAs, miR-125b-5p and miR-223-3p (Figure 4C), were expressed at low levels in LUAD and LUSC (TCGA data); thus, they were selected for further experimental validation.

**Table 1:** The potential targets of POU6F2-AS2.

**Target rank**	**miRNA name**
1	hsa-miR-137
2	hsa-miR-187
3	hsa-miR-223-3p
4	hsa-miR-375
5	hsa-miR-383
6	hsa-miR-125a-5p
7	hsa-miR-125b-5p
8	hsa-miR-351
9	hsa-miR-670
10	hsa-miR-4319

Subsequently, RT-qPCR revealed increased expression of miR-125b-5p in H460 cells following POU6F2-AS2 depletion, whereas transfection with pc-POU6F2-AS2 downregulated miR-125b-5p in SK-MES-1 cells (Figure 4D). These results indicate that miR-125b-5p is sponged by POU6F2-AS2. The sequences predicted to be involved in the binding between POU6F2-AS2 and miR-125b-5p are presented in Figure 4E. Additionally, the luciferase reporter assay confirmed that POU6F2-AS2 directly targeted miR-125b-5p in NSCLC, as indicated by a marked decrease in luciferase activity in miR-125b-5p–overexpressing NSCLC cells cotransfected with wild-type (WT)-POU6F2-AS2 (Figure 4F). Moreover, RNA immunoprecipitation (RIP) assay revealed that POU6F2-AS2 and miR-125b-5p were enriched by the Ago2 antibody in NSCLC cells (Figure 4G), which confirmed that POU6F2-AS2 can function as a ceRNA to adsorb miR-125b-5p. Thus, these findings indicate that POU6F2-AS2 functions as an miR-125b-5p sponge in NSCLC.

### POU6F2-AS2 controls E2F3, a downstream miR-125b-5p target, in NSCLC cells

**Figure 5:**
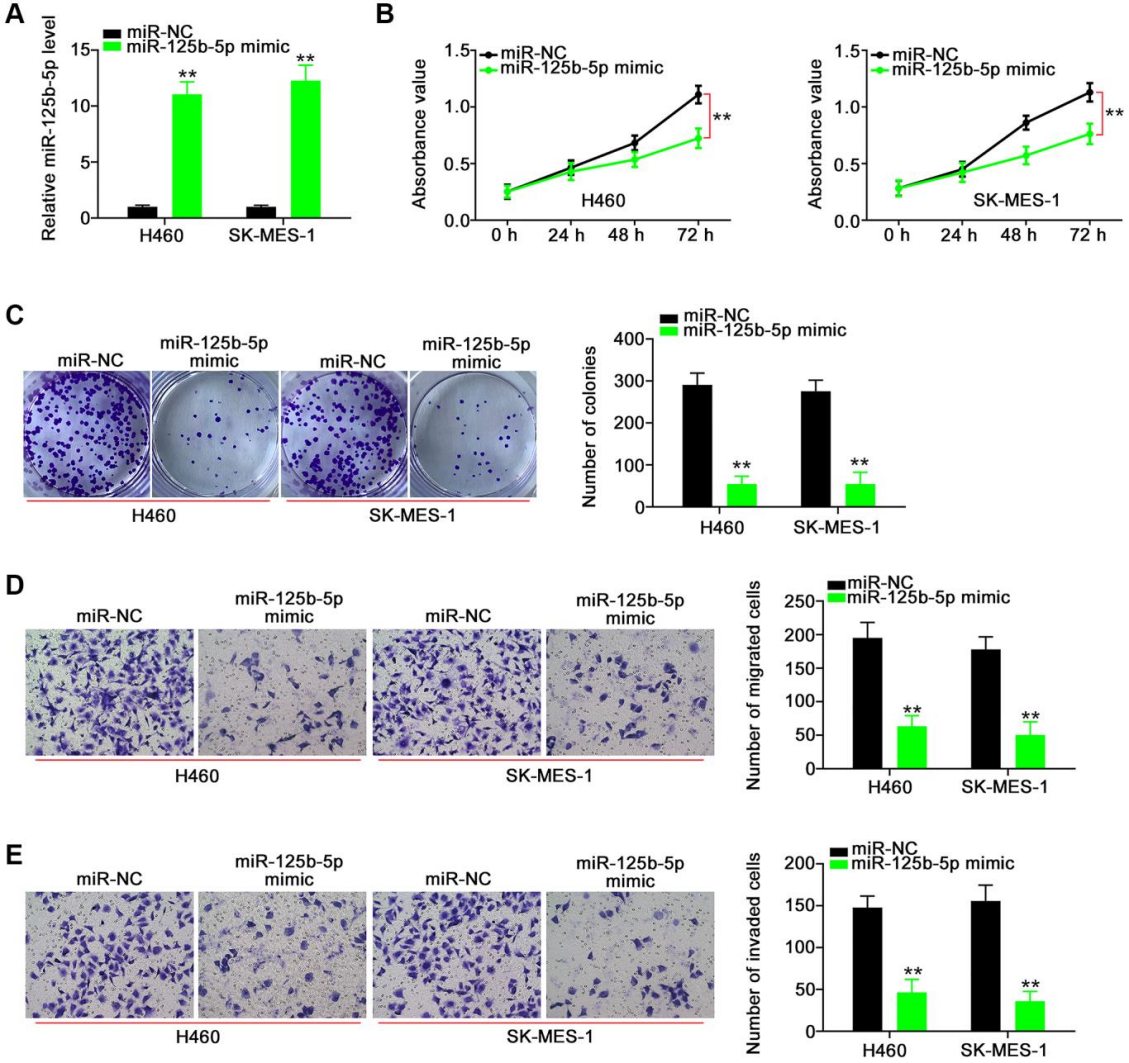
**miR-125b-5p exerts anti-tumor actions in NSCLC cells.** (**A**) The efficiency of miR-125b-5p mimic in NSCLC cells. ^**^P < 0.001 vs. miR-NC group. (**B** and **C**) The proliferation and colony-forming of NSCLC cells after miR-125b-5p overexpression. ^**^P < 0.001 vs. miR-NC group. (**D** and **E**) The motility of NSCLC cells after being transfected with miR-NC or miR-125b-5p mimic. ^**^P < 0.001 vs. miR-NC group.

**Figure 6:**
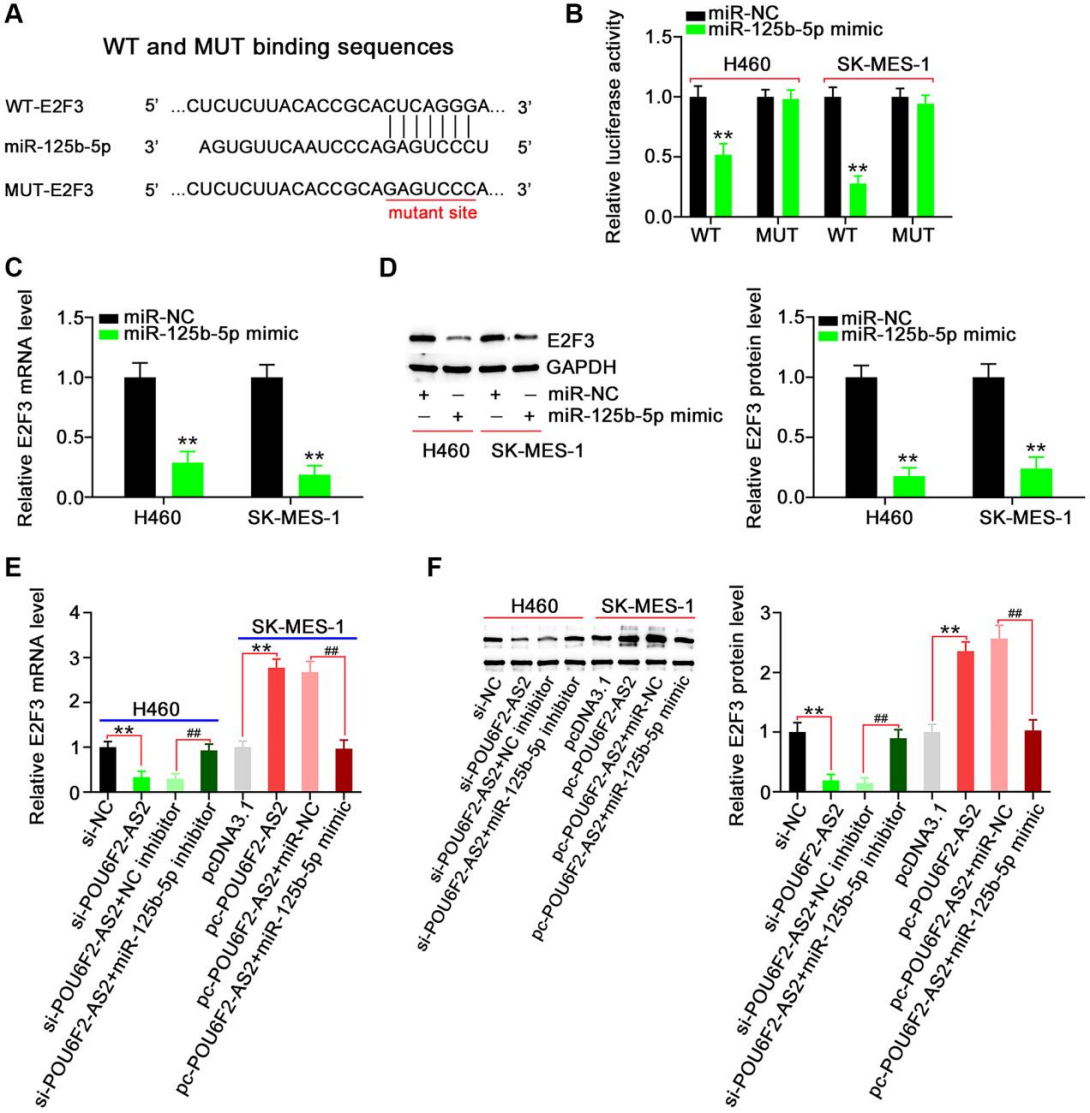
**E2F3, a target of miR-125b-5p, is controlled by POU6F2-AS2 in NSCLC cells.** (**A**) miR-125b-5p possessed binding sequences within E2F3. (**B**) Luciferase activity induced by WT-E2F3 or MUT-E2F3 was examined in NSCLC after miR-125b-5p upregulation. ^**^P < 0.001 vs. miR-NC group. (**C** and **D**) MTDH mRNA and protein levels in miR-125b-5p overexpressed-NSCLC cells. ^**^P < 0.001 vs. miR-NC group. (**E** and **F**) H460 cells were transfected with si-NC, si-POU6F2-AS2, si-POU6F2-AS2+NC inhibitor, or si-POU6F2-AS2+miR-125b-5p inhibitor. SK-MES-1 cells were transfected with pcDNA3.1, pc-POU6F2-AS2, pc-POU6F2-AS2+miR-NC, or pc-POU6F2-AS2+miR-125b-5p mimic. After transfection, the quantification of E2F3 levels was conducted. ^**^P < 0.001 vs. si-NC and pcDNA3.1 groups. ^##^*P* < 0.001 vs. si-POU6F2-AS2+NC inhibitor and pc-POU6F2-AS2+miR-NC groups.

Next, we aimed to address the effect of POU6F2-AS2–miR-125b-5p interaction in NSCLC, for which we first assessed the efficiency of miR-125b-5p mimic (Figure 5A). Functional experiments revealed that the overexpression of miR-125b-5p inhibited the proliferative (Figure 5B), colony-forming (Figure 5C), migratory (Figure 5D), and invasive (Figure 5E) abilities of NSCLC cells. While searching for the putative targets of miR-125b-5p using a bioinformatics approach, we identified a potential binding site between miR-125b-5p and E2F3 (Figure 6A), which was selected for the subsequent experiments to determine the role of E2F3 in affecting NSCLC malignancy [22, 23]. Subsequently, to confirm the biophysical binding between miR-125b-5p and E2F3, we performed luciferase reporter assay, which revealed that the WT-E2F3–induced luciferase activity was downregulated by miR-125b-5p mimic, whereas almost no change was observed in the control mutant (MUT)-E2F3 (Figure 6B). Furthermore, the presence of miR-125b-5p mimic evidently decreased the expression levels of E2F3 in NSCLC cells (Figure 6C and 6D).

After identifying E2F3 as a downstream target of miR-125b-5p, we attempted to explore whether E2F3 was under the regulation of POU6F2-AS2. Notably, the expression levels of E2F3 were suppressed in POU6F2-AS2–depleted H460 cells, whereas it was remarkably increased in pc-POU6F2-AS2–transfected SK-MES-1 cells (Figure 6E and 6F). Furthermore, we observed that coexpression with miR-125b-5p inhibitor rescued the inhibitory effect of si-POU6F2-AS2 on E2F3 in H460 cells. Moreover, miR-125b-5p mimic restored the expression levels of E2F3 to normal, which was elevated by pc-POU6F2-AS2 overexpression in SK-MES-1 cells (Figure 6E and 6F). These findings suggest that POU6F2-AS2 decoys miR-125b-5p in NSCLC cells, thereby regulating E2F3 expression levels.

### Regulatory activities of POU6F2-AS2 in NSCLC are dependent on the miR-125b-5p/E2F3 axis

**Figure 7:**
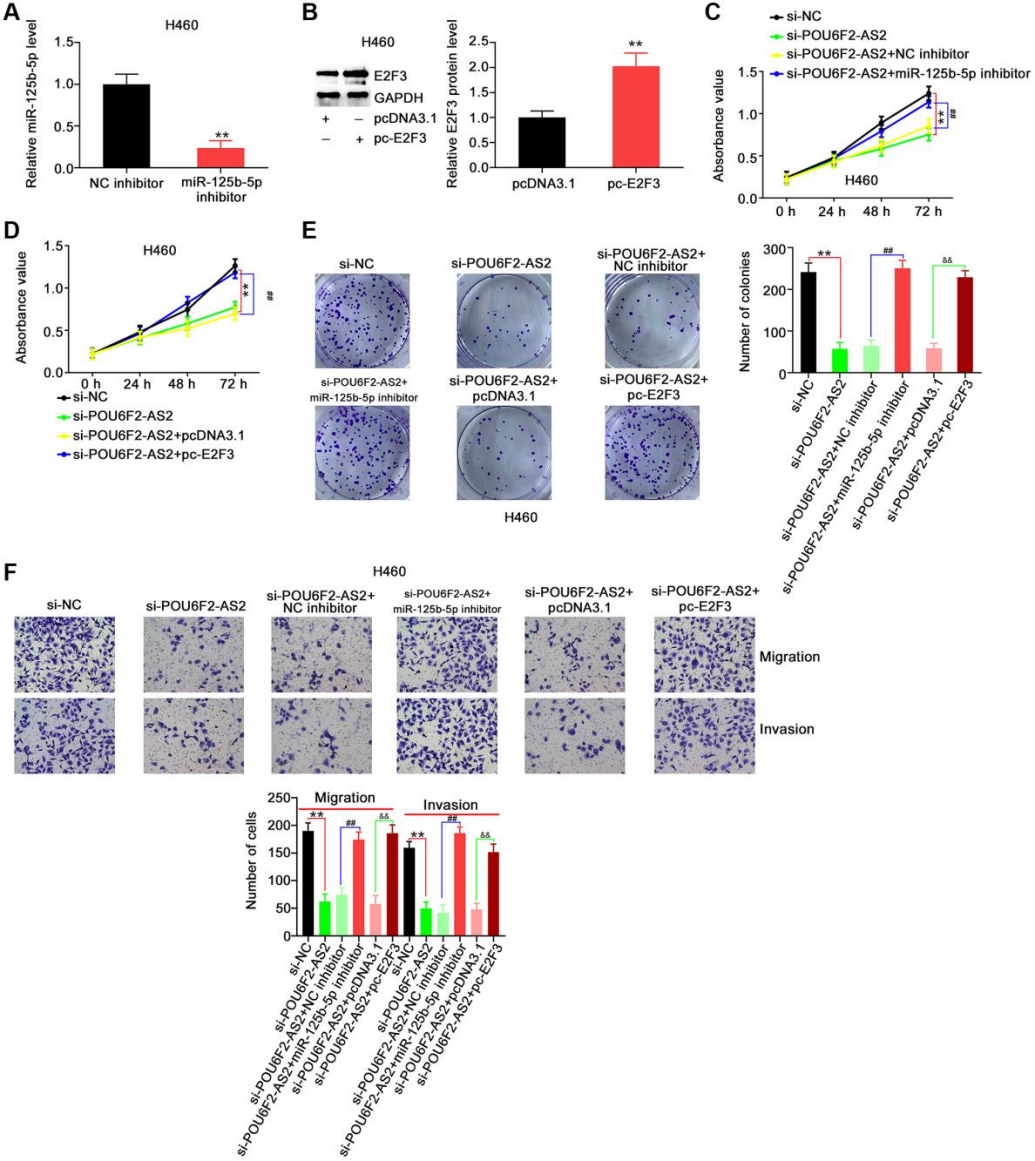
**miR-125b-5p inhibition or E2F3 upregulation reverses the repressing effects of si-POU6F2-AS2 in NSCLC cells.** (**A**) The interference efficiency of miR-125b-5p inhibitor in H460 cells. ^**^P < 0.001 vs. NC inhibitor group. (**B**) The transfection efficiency of pc-E2F3 in H460 cells by western blotting. ^**^P < 0.001 vs. pcDNA3.1 group. (**C**–**E**) H460 cells were transfected with si-NC, si-POU6F2-AS2, si-POU6F2-AS2+NC inhibitor, si-POU6F2-AS2+miR-125b-5p inhibitor, si-POU6F2-AS2+pcDNA3.1, or si-POU6F2-AS2+pc-E2F3. The capacities of proliferative and colony formation were, respectively, measured by CCK-8 and colony formation assays. (**F**) Transwell migration and invasion assays of the motility of H460 cells treated as abovementioned. ^**^P < 0.001 vs. si-NC group; ^##^*P* < 0.001 vs. si-POU6F2-AS2+NC inhibitor group; ^&&^*P* < 0.001 vs. si-POU6F2-AS2+pc-E2F3 group.

To explore whether the miR-125b-5p/E2F3 axis was involved in the function of POU6F2-AS2 in NSCLC cells, we performed rescue experiments. First, we examined the efficiency of miR-125b-5p inhibitor and pc-E2F3 in H460 cells (Figure 7A and 7B), following which they were transfected with si-POU6F2-AS2 alongside miR-125b-5p inhibitor or pc-E2F3. Cell growth analysis using CCK-8 and colony formation assays revealed that the decreased H460 proliferation due to si-POU6F2-AS2 was restored following miR-125b-5p inhibitor or pc-E2F3 treatment (Figure 7C–7E). Additionally, the depletion of POU6F2-AS2 expression levels impeded the motility of H460 cells, and this inhibitory effect was reversed by downregulating the expression of miR-125b-5p or upregulating that of E2F3 (Figure 7F).

**Figure 8:**
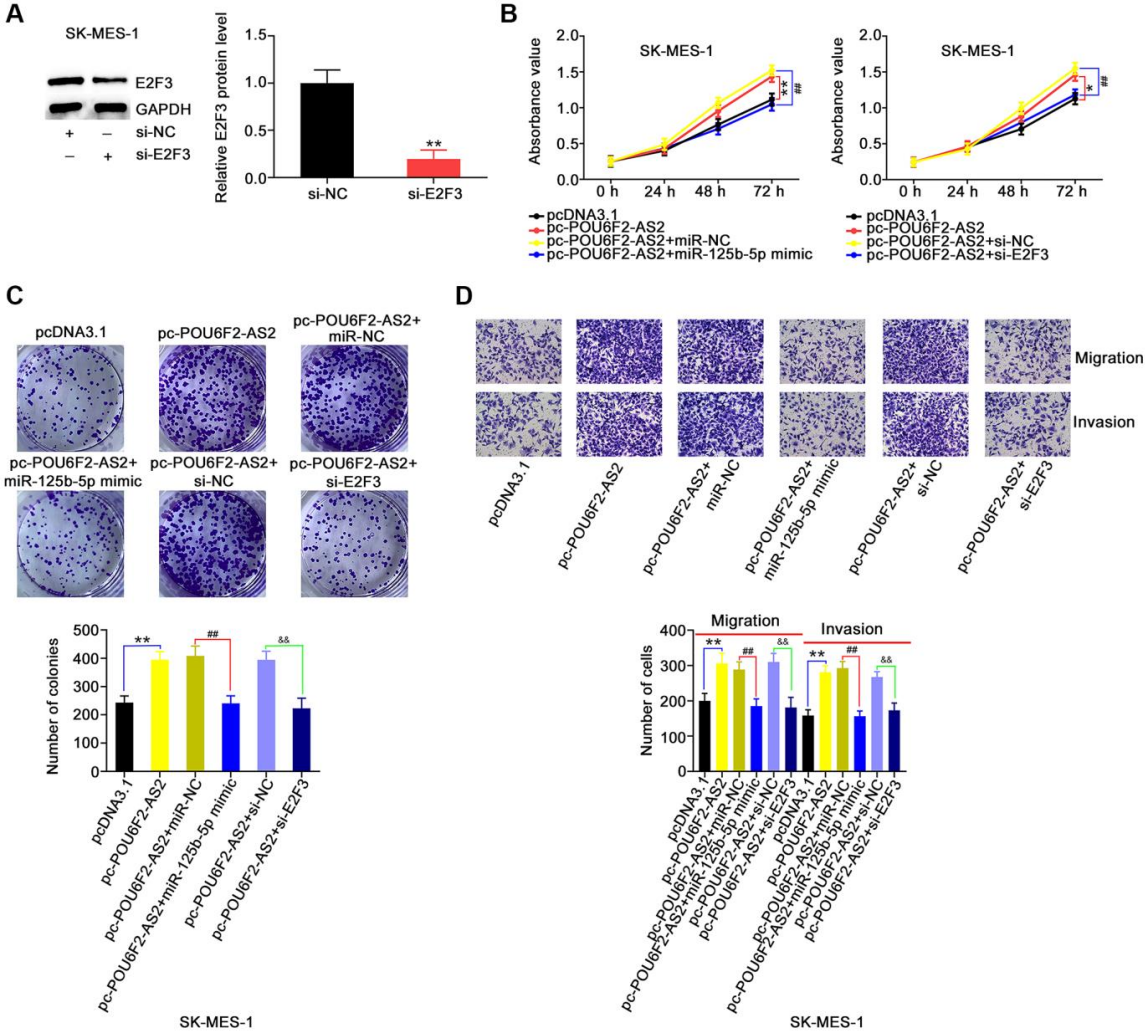
**miR-125b-5p overexpression or E2F3 knockdown counteracts the actions triggered by pc-POU6F2-AS2 in NSCLC cells.** (**A**) E2F3 level in SK-MES-1 cells after pc-E2F3 or pcDNA3.1 transfection. ^**^P < 0.001 vs. si-NC group. (**B** and **C**) POU6F2-AS2-overexpressed SK-MES-1 cells were treated with miR-125b-5p mimic or pc-E2F3. The assessment of cell proliferation and colony formation was implemented applying CCK-8 and colony formation assays, respectively. ^*^P < 0.01 and ^**^P < 0.001 vs. pcDNA3.1 group. ^##^*P* < 0.001 vs. pc-POU6F2-AS2+miR-NC and pc-POU6F2-AS2+si-NC groups. (**D**) Transwell migration and invasion assays were operated to measure cell motility in abovementioned cells. ^**^P < 0.001 vs. si-NC group. ^##^*P* < 0.001 vs. pc-POU6F2-AS2+miR-NC group. ^&&^P < 0.001 vs. pc-POU6F2-AS2+si-NC group.

Furthermore, POU6F2-AS2–overexpressing SK-MES-1 cells were cotransfected with miR-125b-5p mimic or si-E2F3, following the determination of adequate si-E2F3 transfection efficiency (Figure 8A). The upregulation of miR-125b-5p or downregulation of E2F3 abrogated the pro-proliferative actions of pc-POU6F2-AS2 in SK-MES-1 cells (Figure 8B and 8C). Additionally, the effects of pc-POU6F2-AS2 on the motility of SK-MES-1 cells were reversed in the presence of miR-125b-5p mimic or si-E2F3 (Figure 8D). Hence, we concluded that POU6F2-AS2 exacerbated the oncogenicity of NSCLC via the miR-125b-5p/E2F3 pathway.

### POU6F2-AS2 ablation inhibits tumor growth *in vivo*

**Figure 9:**
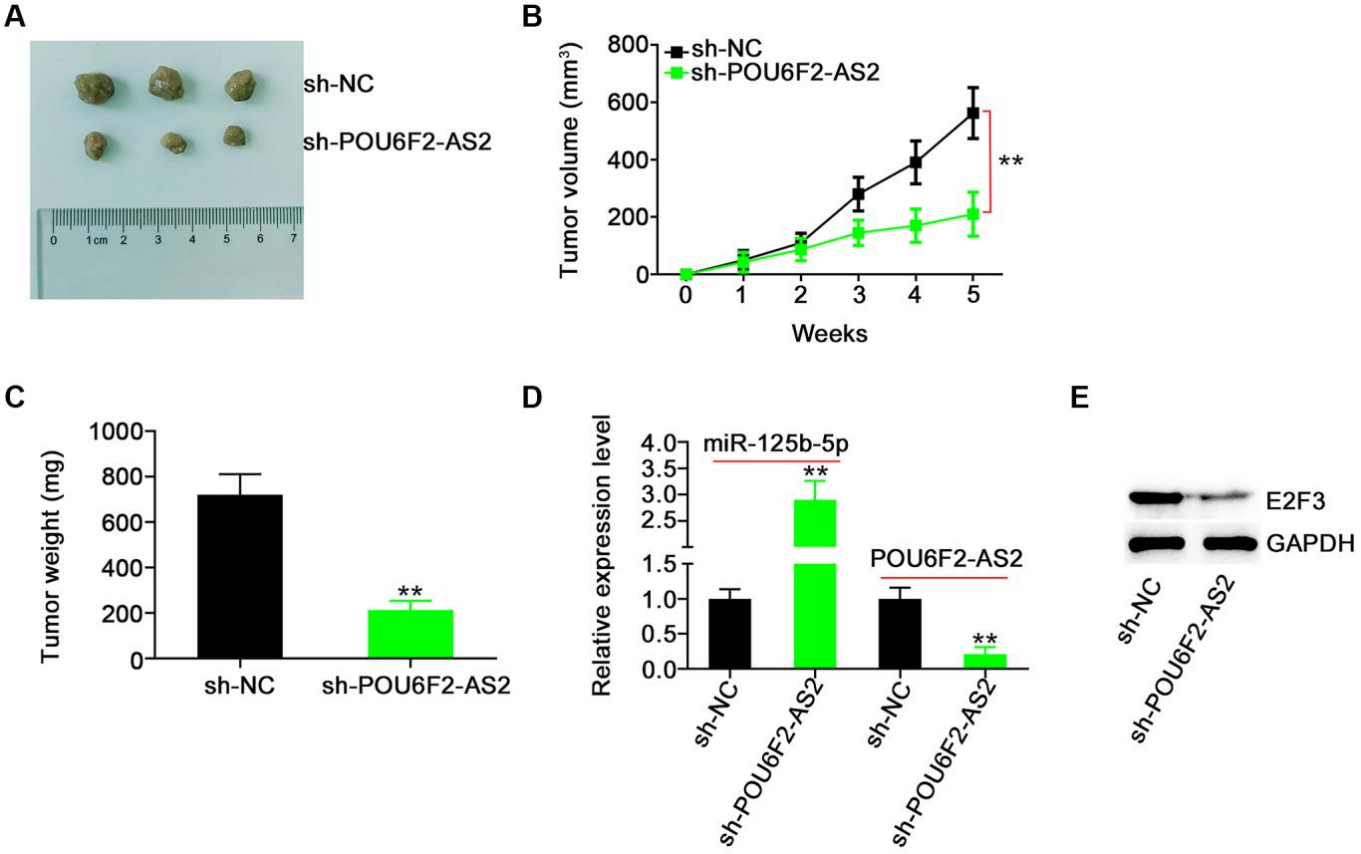
**POU6F2-AS2 downregulation represses tumor growth *in vivo*.** (**A**) The representative image of xenografted tumor tissues. (**B** and **C**) The growth curves and weight of xenografted tumor tissues. ^**^P < 0.001 vs. sh-NC group. (**D**) miR-125b-5p and POU6F2-AS2 levels in xenografted tumor tissues. ^**^P < 0.001 vs. sh-NC group. (**E**) E2F3 protein level in xenografted tumor tissues. ^**^P < 0.001 vs. sh-NC group.

To analyze whether POU6F2-AS2 ablation impaired NSCLC tumor growth, we performed a tumor xenograft experiment. The tumors of the sh-POU6F2-AS2 group exhibited reduced tumor growth (Figure 9A and 9B) and weight (Figure 9C) compared with those of the control sh-negative control (NC) group. Additionally, miR-125b-5p was overexpressed in POU6F2-AS2–depleted tumor xenografts (Figure 9D). Furthermore, POU6F2-AS2 was ablated in tumor xenografts following stable sh-POU6F2-AS2 transfection. Moreover, the E2F3 expression levels were decreased in tumor xenografts of the sh-POU6F2-AS2 group (Figure 9E). Thus, POU6F2-AS2 inhibition suppressed the growth of NSCLC tumors *in vivo*.

## DISCUSSION

Recently, numerous studies have reported that the pathogenesis of NSCLC is controlled by both protein-coding and nonprotein-coding genes [24, 25]. The aberrant expression levels of lncRNAs markedly affect the onset and development of NSCLC by regulating multiple pathological behaviors [26, 27]. Therefore, determining the specific functions and underlying molecular events of lncRNAs in NSCLC can help identify novel diagnostic and therapeutic targets for this disease. However, to the best of our knowledge, there has been no systematic clarification regarding the contribution of several identified lncRNAs to the progression of NSCLC, thereby necessitating further research. Herein, we demonstrated that POU6F2-AS2 exerted pro-oncogenic activities in NSCLC by targeting the miR-125b-5p/E2F3 axis.

Recently, several lncRNAs have been reported to be closely associated with NSCLC progression [12, 28]. For example, the lncRNAs PLAC2 [29], NUBE2R2-AS1 [30], and OXCT1-AS1 [31], which are upregulated in NSCLC, promote tumor oncogenicity. Conversely, the lncRNAs HAR1A [32], LSAMP-1 [33], and LINC00174 [34] were underexpressed in NSCLC and inhibited cancer aggressiveness. Our literature survey revealed that studies focused on the expression status and detailed roles of POU6F2-AS2 in NSCLC are not extant. Herein, a considerable upregulation of POU6F2- AS2 in NSCLC tissues and cell lines was confirmed. Moreover, while POU6F2-AS2 inhibition decreased NSCLC cell proliferation, colony formation, and motility, POU6F2-AS2 upregulation demonstrated the opposite effects. Overall, these results improve our understanding of the complicated molecular events underlying NSCLC genesis and progression.

Mechanistically, although lncRNAs possess no protein-coding potential, they can affect gene expression through diverse mechanisms [35]. LncRNAs can interact with different entities, such as miRNAs, mRNAs, proteins, or DNAs, and subsequently alter their distribution and expression levels, thereby influencing their roles [36]. The mechanisms by which lncRNAs exert their effects are chiefly determined by their subcellular localization [37]. Accordingly, we determined the location of POU6F2-AS2 in NSCLC cells and found that it is primarily distributed in the cytoplasm. The cytoplasmic presence of lncRNAs has attracted substantial research, consequently establishing the ceRNA theory, which suggests that lncRNAs can sequester miRNAs through the miRNA response element and suppress the effect of miRNA functions on their targets [38].

To further explain the action mechanism of POU6F2-AS2, bioinformatics prediction was performed, which revealed that miR-125b-5p harbors complementary sequences that can pair with POU6F2-AS2, which was subsequently confirmed via luciferase reporter and RIP assays. Furthermore, when the downstream target of miR-125b-5p in NSCLC was explored, E2F3 was identified as a direct target of the miRNA. Notably, E2F3 was positively regulated by the decoy action of POU6F2-AS2 for miR-125b-5p. Thus, POU6F2-AS2 acts as an endogenous decoy for miR-125b-5p in NSCLC, thereby modulating E2F3 expression levels. These results indicate that POU6F2-AS2, miR-125b-5p, and E2F3 RNAs constitute a novel ceRNA pathway in NSCLC.

The role of miR-125b-5p has been well studied in human cancers and is reportedly downregulated in laryngeal squamous cell carcinoma [39], breast cancer [40], hepatocellular carcinoma [41] and ovarian cancer [42]. Furthermore, miR-125b-5p is reportedly expressed at low levels in LUAD, which was associated with poor prognosis [43]. Additionally, serum miR-125b-5p was found to be remarkably correlated with NSCLC stage and prognosis [44]. Multivariate analysis revealed that the elevated expression levels of miR-125b-5p are an independent prognostic factor for survival [44]. Herein, miR-125b-5p downregulation in NSCLC was confirmed. Functionally, miR-125b-5p was involved in modulating numerous malignant properties in NSCLC cells. Mechanistically, multiple targets of miR-125b-5p have been identified, including HK2 [39], KIAA1522 [40], TXNRD1 [41], CD147 [42], VEGFA [45], and TNFR2 [46]. We discovered E2F3, a key regulator of the G1/S phase transition, as a downstream target of miR-125b-5p in NSCLC. Earlier, E2F3 expression was reportedly increased in NSCLC and closely associated with early lymphatic spread [22]. Moreover, E2F3 expression was confirmed to be an independent factor for predicting patient prognosis in NSCLC [22]. Via rescue experiments, we have further proved that suppressing miR-125b-5p or increasing E2F3 expression levels sufficiently recovered POU6F2-AS2 depletion–induced anticarcinostatic activities in NSCLC. Briefly, the miR-125b-5p/E2F3 axis constitutes the downstream effector of POU6F2-AS2 and influences the aggressiveness of NSCLC. However, we did not explore the regulation of NSCLC metastasis *in vivo* by POU6F2-AS2 or the factors that caused the aberrant expression of POU6F2-AS2 in NSCLC; therefore, further investigations are warranted.

In conclusion, we have found that the expression levels of POU6F2-AS2 were notably elevated in NSCLC, potentially aggravating its oncogenicity and serving as a molecular sponge for miR-125b-5p through the ceRNA pattern, consequently upregulating E2F3 expression levels. Our findings indicate that POU6F2-AS2 is an attractive diagnostic biomarker and therapeutic target for NSCLC.

## MATERIALS AND METHODS

### Clinical specimens

This study was approved by the Ethics Committee of The People’s Hospital of Liaoning Province. All patients provided written informed consent before the implementation of the study. The study included 47 patients with NSCLC from whom NSCLC and adjacent nontumorous tissues were collected. The inclusion criteria were as follows: (1) patients who were diagnosed with NSCLC; (2) patients who had not been treated with radiotherapy, chemotherapy, or immunotherapy; and (3) patients who had not been diagnosed with other human cancers. The exclusion criteria were as follows: (1) patients who had received radiotherapy or chemotherapy; (2) patients who received immunotherapy; and (3) patients who presented with other cancers. All clinical specimens were immersed in liquid nitrogen following collection until further use.

### Cell lines and transfection

The human nontumorigenic bronchial epithelial cell line BEAS-2B (ATCC, Manassas, VA, USA) was maintained in bronchial epithelial cell growth media (Lonza, Walkersville, MD, USA). The NSCLC cell lines A549, SK-MES-1, and H460 were cultured in RPMI-1640, F-12K (Gibco, Thermo Fisher Scientific, Inc.), and Minimum Essential Medium (Gibco) media, respectively. All the NSCLC cell lines were purchased from ATCC. Additionally, 10% fetal bovine serum (FBS) and 1% penicillin/streptomycin mixed reagent (Gibco) were added to the culture media. The aforementioned cells were maintained at 37°C and grown in a humidified incubator with 5% CO_2_ atmosphere.

Three siRNAs against POU6F2-AS2 (si-POU6F2-AS2) expression, NC siRNA (si-NC), E2F3 siRNA (si-E2F3), POU6F2-AS2 overexpression vector (pc-POU6F2-AS2), and E2F3 overexpression vector pcDNA3.1-E2F3 (pc-E2F3), were obtained from GenePharma Company (Shanghai, China). miR-125b-5p mimic, miRNA NC mimic (miR-NC), miR-125b-5p inhibitor, and NC inhibitor were obtained from RiboBio Co., Ltd. (Guangzhou, China). Transient transfection was performed using Lipofectamine 2000 (Invitrogen).

### Quantitative real-time polymerase chain reaction

The total RNA was extracted from tissues or cells using TRIzol (Takara, Dalian, China). To determine POU6F2-AS2 and E2F3 expression levels, the total RNA was reverse-transcribed into complementary DNA using PrimeScript™ RT reagent kit (Takara). Next, PCR amplification was performed using TB Green^®^ Premix Ex Taq™ II (Takara). Glyceraldehyde 3-phosphate dehydrogenase (GAPDH) was used for normalization. To quantify miR-125b-5p expression levels, reverse transcription and PCR were performed using miRcute miRNA First-Strand cDNA Synthesis Kit and miRcute miRNA qPCR Detection Kit SYBR Green (Tiangen Biotech, Beijing, China), respectively. U6 was used as the internal control to analyze miR-125b-5p expression levels. We used the 2^−ΔΔCq^ method to calculate gene expression levels.

### Colony formation assay

The cells were harvested 24 h following transfection. Cell suspension (2 mL) containing 500 cells were layered on the wells of six-well plates. On day 15, the culture media was discarded, and the newly formed colonies were fixed with methanol and stained with crystal violet. After extensive washing, the colonies were imaged and counted under a light microscope (Olympus, Tokyo, Japan).

### Cell counting Kit-8 assay

The transfected cells were harvested and seeded onto 96-well plates with an initial density of 2 × 10^3^ cells/well. After culturing for different periods, cell proliferation was detected via incubation with 10 μL CCK-8 solution (Dojindo, Kumamoto, Japan) at 37°C for 2 h, following which the optical density value was determined by measuring the absorbance at 450 nm using a microplate reader.

### Transwell migration and invasion assays

For the migration assay, 5 × 10^4^ cells diluted in 200 μL basal media without FBS were added into the apical chambers (Corning Costar, Cambridge, MA, USA). The transwell basolateral chambers were loaded with 700 μL media culture supplemented with 10% FBS. After culturing for 24 h, the non-migrated cells were washed away, and the migrated cells were fixed and stained with 4% paraformaldehyde and crystal violet, respectively. The stained cells were imaged and counted under a light microscope. For the invasion assay, Matrigel (BD Bioscience) was coated on the upper side of the membranes, and the rest of the procedure was performed same as mentioned above.

### Tumor xenograft experiment

Short-hairpin RNAs (shRNAs) against POU6F2-AS2 (sh-POU6F2-AS2) and NC shRNA (sh-NC) were designed and synthesized by GenePharma and inserted into the pLKO.1 vector (Addgene, Inc.). The final construct was transfected into 293T cells. After 2 days of incubation, the lentivirus was transduced into H460 cells. H460 cells with stable POU6F2-AS2 knockdown were selected using puromycin.

Animal experiments were performed with approval from the Institutional Animal Care and Use Committee of The People’s Hospital of Liaoning Province. Male BALB/c nude mice (SLAC Laboratory Animal Co., Ltd., Shanghai, China) aged 4–6 weeks were housed under specific pathogen-free conditions at 25°C and 50% humidity, with a 10:14 light/dark cycle and *ad libitum* access to food and water. The mice were injected with 2 × 10^6^ H460 cells with the stable expression of sh-POU6F2-AS2 or sh-NC. Each group had three mice. After tumor cell injection, the size of the tumor xenografts was monitored weekly using a caliper. On day 35, all mice were euthanized, and subcutaneous xenografts were collected and weighed.

### Nuclear–cytoplasmic fractionation assay

To assess the cellular localization of POU6F2-AS2 in NSCLC cells, Cytoplasmic and Nuclear RNA Purification Kit (Norgen, Thorold, ON, Canada) was used. After extracting POU6F2-AS2 from the cytoplasm and nucleus, RT-qPCR was performed to determine its expression levels in NSCLC cells.

### Bioinformatics prediction

TCGA database (https://tcga-data.nci.nih.gov/tcga/) was used to analyze the expression of POU6F2-AS2 in LUAD and LUSC. lncLocator (http://www.csbio.sjtu.edu.cn/bioinf/lncLocator/) was used to predict the location of POU6F2-AS2. miRcode (http://www.mircode.org/) was used to predict the binding between POU6F2-AS2 and miR-125b-5p. TargetScan (http://www.targetscan.org) and Starbase3.0 (https://starbase.sysu.edu.cn/) were employed to determine the downstream target of miR-125b-5p.

### Luciferase reporter assay

The POU6F2-AS2 fragment containing the WT miR-125b-5p binding site was synthesized by GenePharma and cloned into the psiCHECK™-2 vector (Promega). The generated luciferase reporter vector was designated as WT-POU6F2-AS2. Meanwhile, the luciferase reporter vectors, namely MUT-POU6F2-AS2, WT-E2F3, and MUT-E2F3, were synthesized and used for the subsequent experiments. WT and MUT alongside miR-125b-5p mimic or miR-NC were cotransfected into NSCLC ells. The transfected cells were collected and their luciferase activity was measured using a dual-luciferase reporter assay system (Promega).

### RNA immunoprecipitation

RIP was performed using EZ-Magna RIP™ RNA Binding Protein Immunoprecipitation Kit (Millipore, Billerica, MA, USA). Cell lysates were produced by cultivating NSCLC cells with complete RIP buffer. Next, 100 μL cell lysate was treated with magnetic beads conjugated with anti-Ago2 or control IgG (Millipore). Following overnight incubation at 4°C, the magnetic beads were collected and subjected to Proteinase K buffer for protein digestion. The immunoprecipitated RNA was extracted and analyzed using RT-qPCR.

### Western blotting

The total protein was extracted using RIPA buffer (Beyotime, Shanghai, China) supplemented with phosphatase and protease inhibitors (KeyGEN BioTECH; Nanjing, China) and quantified using Enhanced BCA Protein Assay Kit (Beyotime). Subsequently, 10% sodium dodecyl sulfate–polyacrylamide gel electrophoresis was used to separate equal amounts of proteins, followed by their transfer onto a polyvinylidene fluoride (PVDF) membrane. Nonspecific proteins were blocked with 5% skim milk at room temperature for 2 h. After overnight incubation with a primary antibody, anti-E2F3 (1:1,000 dilution; ab152126) or anti-GAPDH (1:1,000 dilution; ab128915; Abcam, Cambridge, UK), the PVDF membrane was incubated with a secondary antibody (Abcam) for 2 h. Protein blots were subsequently visualized using BeyoECL Plus Kit (Beyotime).

### Statistical analysis

The results from three independent experiments were expressed as mean ± standard deviation. The treatment groups were compared using paired and unpaired Student’s *t*-test (two groups) or one-way analysis of variance followed by Duncan’s test (multiple groups). A *p* value of <0.05 was considered statistically significant.

### Availability of data and materials

Data are available with the communication author and can be provided upon reasonable request.
